# Outcome and treatment-related adverse events of combined immune checkpoint inhibition with flipped dosing in a real-world cohort of 79 patients with metastasized melanoma

**DOI:** 10.3389/fonc.2023.1256800

**Published:** 2023-11-30

**Authors:** Charlotte Nübel, Teresa Amaral, Ulrike Leiter, Lukas Flatz, Andrea Forschner

**Affiliations:** Department of Dermatology, University Hospital of Tübingen, Tübingen, Germany

**Keywords:** combined immune checkpoint inhibitors, immune related adverse events, advanced melanoma, immunotherapies, nivolumab, ipilimumab

## Abstract

**Introduction:**

Combined immune checkpoint inhibition (ICI) with ipilimumab and nivolumab is a widely used treatment regimen for metastatic melanoma with non-resectable metastases. Nevertheless, the standard dose of ipilimumab 3 mg/kg bw and nivolumab 1 mg/kg bw is associated with a high rate of treatment-related adverse events (trAEs) (59% grade 3–4). In the CheckMate 511 study, it could be shown that flipped dosing with ipilimumab 1 mg/kg bw and nivolumab 3 mg/kg bw resulted in a significant reduction of trAE.

**Methods:**

We have also used this regimen in the clinical setting and report the trAE, progression-free survival, and overall survival for 79 patients with metastatic melanoma who started combined ICI in the flipped dosing between March 2019 and April 2020.

**Results:**

in total, 40 patients started first-line, 50% of whom had an elevated lactate dehydrogenase level at baseline. The disease control rate of these patients was 50%. The 2-year overall survival rate 67%. Moreover, 33% of the patients suffered grade 3 or 4 treatment related adverse events.

**Discussion:**

The results of our study correspond very well to the results of the CheckMate 511 study (2-year OS: 65%, grade 3-4 immune-related side effects: 35%). Combined ICI with ipilimumab 1 mg/kg bw and nivolumab 3 mg/kg bw seems to be an equally effective but better-tolerated therapy regimen for metastasized melanoma patients, also in a real-world cohort.

## 1 Introduction

Immune checkpoint inhibitors (ICI) like ipilimumab (a monoclonal antibody against cytotoxic T-lymphocyte antigen 4) and nivolumab (monoclonal antibody against programmed cell death 1) have changed the treatment landscape of advanced melanoma in the last decade (1). In the CheckMate 067 study, 59% of adverse events grades 3 and 4 occurred in the combined ICI arm with nivolumab 1 mg/kg bw and ipilimumab 3 mg/kg bw. In the nivolumab monotherapy arm, the rate of treatment-related adverse events (trAEs) was 21% and in the ipilimumab monotherapy arm 28%. The median overall survival was 72 months after a minimum follow-up of 6.5 years for the combined ICI arm, 37 months for the nivolumab, and 20 months for ipilimumab monotherapy arm (1–3). Despite the high efficacy, many patients suffer trAE such as hepatitis, colitis, pneumonitis, or endocrinological side effects (4). The clinical symptoms of trAE can be unspecific and hard to detect; on the other side, the immediate treatment of trAE can be lifesaving (5). In the literature, trAEs are described in approximately 60% of the patients with combined ICI (6–9).

In the CheckMate 511 study, the flipped dosing with ipilimumab 1 mg/kg bw and nivolumab 3 mg/kg bw resulted in a significant reduction of trAE without significant differences in progression-free survival (PFS) and overall survival (OS) between the two cohorts. The median PFS was 9.9 months in the flipped dose regime (nivolumab 3 mg/kg bw and ipilimumab 1 mg/kw bw), and the grades 3 and 4 trAE was approximately 34% compared to 8.9 months in the standard dosing (nivolumab 1 mg/kg bw and ipilimumab 3 mg/kg bw) and 48% of adverse events (10). The flipped dosing with ipilimumab 1 mg/kg bw and nivolumab 3 mg/kg bw was associated with a significant reduction in trAE (10). Nevertheless, the CheckMate 511 study included only patients that met the inclusion criteria (e.g., no active brain metastasis, no uveal melanoma, ECOG 0 or 1), which does not represent our daily clinical setting.

In the following study, the aim was to evaluate the efficacy, which means response rates, progression-free and overall survival as well as frequency of trAE in melanoma patients receiving ipilimumab 1 mg/kg bw plus nivolumab 3 mg/kg bw in daily clinical routine.

## 2 Materials and methods

In this study, all advanced melanoma patients who started combined ICI with ipilimumab (1 mg/kg bw) and nivolumab (3 mg/kg bw) at the University Dermatological Clinic of Tübingen in the period from March 2019 to April 2020 were included. The initial tumor stage was evaluated at the beginning of treatment in accordance with the 8th edition of the American Joint Commission on Cancer (AJCC) staging manual. The cutoff date for evaluating the data was at least 24 months after the combined ICI treatment initiation (April 2022).

The baseline and follow-up data as well as clinical characteristics were collected from the patients’ electronic records. The baseline data included sex, age, type of melanoma and date at initial diagnosis, initiation of therapy, and the reasons for choosing the flipped dose instead of the standard dose. Furthermore, histological tumor type and mutation status were recorded. Data on laboratory parameters such as tumor marker Protein S100, lactate dehydrogenase (LDH) level, and differential blood count were collected. The type and number of metastases, history of autoimmune diseases, and follow-up therapies after combined ICI were documented. In addition, it was assessed whether the patients had already been pre-treated before or whether combined ICI was initiated in a first-line setting. The tumor response was evaluated based on the clinical and radiologic reports after approximately 12 weeks of the initiation of therapy. The response to ICI was classified according to the revised Response Evaluation Criteria in Solid Tumors (RECIST) guidelines (version 1.1) with complete response (CR), partial response (PR), stable disease (SD), or progressive disease (PD) as possible outcomes. The overall response rate was defined as the sum of CR and PR. The disease control rate was defined as the sum of CR, PR, and SD. All cases were discussed before the initiation of combined ICI and again with the staging results in the interdisciplinary tumor board of the University of Tübingen. The patients received staging with brain, neck, thoracic, abdomen, and pelvic computed tomography scheduled every 3 months. Treatment-related adverse events were classified according to the National Cancer Institute Common Terminology Criteria for Adverse Events (CTCAE) (version 4.0) based on the patient’s electronic file. We considered trAE that had been reported in the CheckMate 511 study and added further trAEs when necessary. Where the patient’s record was not clear enough about the CTCAE grade, we classified it as grade 3 if the patient had received oral corticosteroids (inpatient or outpatient), and we classified it as grade 4 if the patient had to be hospitalized or received intravenous corticosteroids.

We used descriptive statistics for the patients’ characteristics. Overall survival was defined from the beginning of ICI to death or the last date of follow-up. Progression-free survival was calculated as the time from initiation of ICI to death or disease progression or the last date of follow-up. Kaplan–Meier curves and log rank test were used to calculate and visualize potential survival differences. The level of significance was 0.05 (two-sided) in all analyses. Adjustment for multiple testing was not performed. The analyses were performed with SPSS version 27 and STATA version 17. Microsoft Excel Version 16.72 was used to produce tables.

All patients had given their consent for their data to be used for research purposes. Furthermore, approval has been obtained with project number 899/2021BO2 by the Ethics Committee of Eberhard-Karls-University of Tübingen from 14.12.2021.

## 3 Results

### 3.1 Characteristics of all patients

We identified 79 patients with advanced unresectable melanoma who started combined ICI therapy with the flipped dose regime. All of them started with ipilimumab 1 mg/kg bw plus nivolumab 3 mg/kg bw every 3 weeks for normally four doses, followed by nivolumab 480 mg monotherapy afterwards. The baseline patient characteristics of the total cohort are summarized in **Table 1** (left side).

**Table 1:** Baseline patient characteristics total cohort and first-line cohort.

	Total cohort		First-line cohort	
	*N*	%	*N*	%
Total	79	100	40	100
Sex				
Male	46	58.2	18	45.0
Female	33	41.8	22	55.0
Age, median (range)	64.0 (27–91)		66.5 (48–88)	
<65 years	42	53	20	50.0
65–75 years	12	15.2	5	12.5
≥75 years	25	31.6	15	37.5
Melanoma type				
Cutaneous	48	60.8	20	50.0
Ocular	13	16.5	11	27.5
Unknown primary	12	15.2	8	20.0
Mucosal	5	6.3	1	2.5
Acral lentiginous	1	1.2	0	
AJCC cancer stage at study inclusion				
III	2	2.6	1	2.5
IV	77	97.4	39	97.5
BRAF V600 mutation				
Wild type	53	67.1	32	80.0
Mutant	25	31.6	8	20.0
Unknown	1	1.3	0	
Reason for choosing flipped dose				
Side effects with prior ICI	9	11.5		
Patient-oriented or individual risk–benefit assessment	70	88.5	40	100.0
First-line therapy of combined ICI				
Yes	40	50.6	40	100.0
No	39	49.4		
S100 at start of ICI				
S100 elevated	47	59.5	22	55.0
S100 normal	30	38	18	45.0
S100 unknown	2	2.5	0	
LDH at start of ICI				
LDH elevated	44	55.7	20	50.0
LDH normal	33	41.8	18	45.0
LDH unknown	2	2.5	2	5.0
Number of metastatic organ system at start of ICI				
One organ system	4	5.1	3	7.5
Two organ systems	20	25.3	14	35.0
≥3 organ systems	55	69.6	23	57.5
Metastases stage at entry				
M0, M1a, and M1b	13	16.5	8	20.0
M1c	54	68.4	26	65.0
M1d	12	15.2	6	15.0
Four cycles of combined ICI completed				
Yes	42	53.2	23	57.5
No	37	46.8	17	42.5
Treatment-related adverse event during treatment				
No	17	21.5	7	17.5
Yes	62	78.5	33	82.5
First treatment-related adverse event after cycle, mean (range)	1.6 (1–4)		1.71 (1–4)	
1	35	57.4	18	56.3
2	17	27.9	8	25
3	5	8.2	3	9.4
4	4	5.1	3	9.4
Discontinuation after cycle, mean (range)	2.9 (1–3)		3.175 (1–3)	
1	16	43.3	6	35.3
2	11	29.7	4	23.5
3	10	27.0	7	41.1

#### 3.1.1 Efficacy total cohort

**Table 2** (left side) displays the response of the first staging after ICI treatment initiation of the total cohort. The overall response rate was 23%, and the disease control rate was 38%. Seven out of 40 patients had died before staging.

**Table 2:** Response of total cohort (left side) and first-line cohort (right side).

	Total cohort		First-line cohort	
	*N* = 79	%	*N* = 40	%
Complete remission (CR)	4	5.1	4	10.0
Partial remission (PR)	14	17.7	9	22.5
Stable disease (SD)	12	15.2	7	17.5
Progressive disease (PD)	42	53.2	18	45.0
Death before staging	7	8.9	2	5.0

The main reason why 37 patients could not receive all four cycles as scheduled was severe trAE (**Table 3**).

**Table 3:** Reasons for discontinuation total cohort.

	*N* = 37	%
General deterioration of condition	6	16.2
Severe trAE	15	40.5
Died within 30 days after immunotherapy because of progressive disease*	6	16.2
Progressive Disease (before Staging)	4	10.8
Therapy change after mutation analysis	2	5.4
Change therapy regime to Best supportive care	2	5.4
Unknown	2	5.4

However, nearly one-third (32%) of the patients with premature discontinuation of combined ICI were able to continue with nivolumab monotherapy and more than one third (35%) received no further therapy (**Table 4**).

**Table 4:** Subsequent therapy total cohort.

	*N* = 37	%
Nivolumab monotherapy	12	32.4
No following therapy	13	35.1
Chemotherapy	6	16.2
Target therapy	2	5.4
Best supportive care	2	5.4
Unknown	2	5.4

#### 3.1.2 Overall survival total cohort

In the total cohort, the median OS was 18 months (95% CI, 5.5–30.3). The 2-year overall survival was 47% (95% CI, 44.9–48.92). In view of the BRAF mutation status, patients with BRAF V600 mutation had a significantly improved OS compared to patients with BRAF wild-type tumors (*p* < 0.001). The 2-year overall survival was 64% (95% CI, 62.04–65.97) in BRAF V600 mutant patients compared to 40% (95% CI, 37.8–41.7) in the BRAF wild-type cohort.

**Figure 1:**
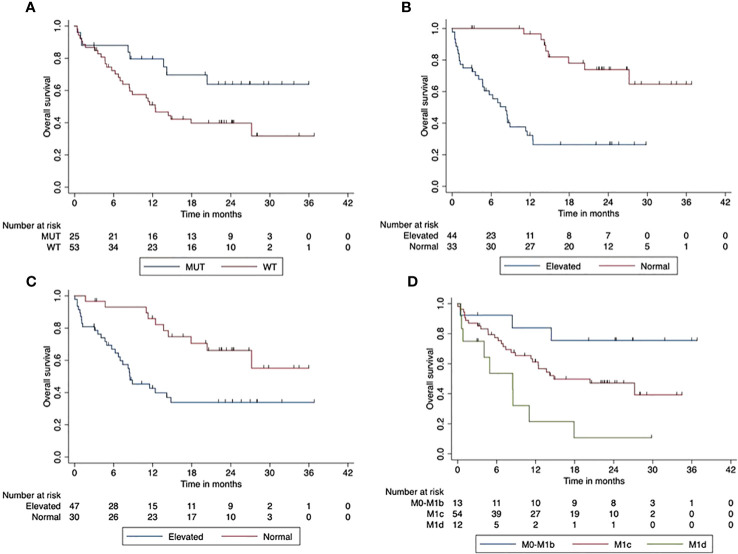
Impact of patient characteristics (total cohort) on overall survival. **(A)** BRAF mutant vs. wild-type tumors (*p* < 0.001). **(B)** LDH at start normal vs. elevated (*p* < 0.001). **(C)** Protein S100 at start normal vs. elevated (*p* = 0.02). **(D)** M-stage at start M0-M1d (*p* = 0.002).

Patients with normal LDH at the start of ICI had a significantly improved OS compared to patients with elevated LDH at baseline (*p* < 0.001). The normal LDH baseline resulted in a 2-year OS of 74% (95% CI, 72.03–75.95). Furthermore, the OS was significantly better with Protein S100 normal at ICI start (*p* = 0.02). The 2-year OS of these patients was 66% (95% CI, 64.3–68.3) compared to 34% (95% CI, 32.0–35.94) in patients with elevated S100. Higher AJCC stages had a significantly negative impact on OS (*p* = 0.002). **Figure 1** shows the impact of patients’ characteristics on overall survival.

#### 3.1.3 Progression-free survival total cohort

**Figure 2:**
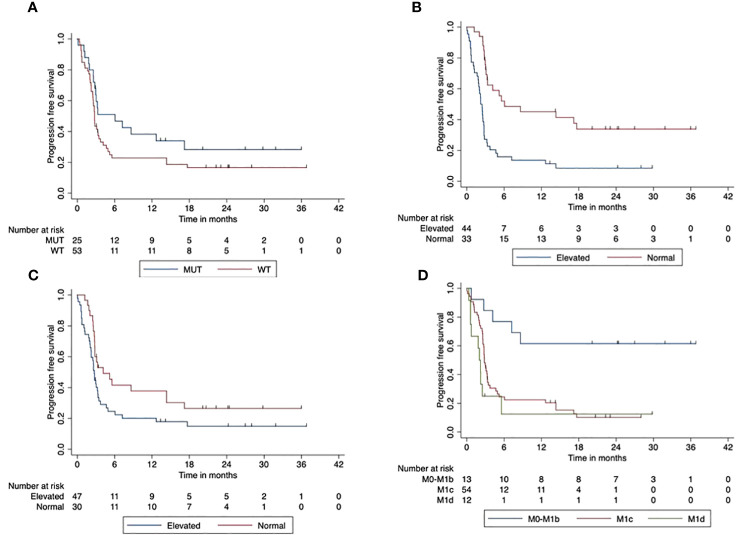
Impact of patient characteristics (total cohort) on progression-free survival. **(A)** BRAF mutant vs. wild-type tumors (*p* < 0.001). **(B)** LDH at start normal vs. elevated (*p* < 0.001). **(C)** Protein S100 at start normal vs. elevated (*p* = 0.047). **(D)** M-stage at start (*p* < 0.001).

The median PFS of the total cohort was 3 months (95% CI, 2.6–3.4). The 2-year PFS was 20% (95 CI%, 0.09–4.0). We found improved PFS for patients with BRAF V600 mutant tumors compared to BRAF wild-type tumors (*p* = 0.047). The 2-year PFS was 17% (95% CI, 14.7–18.6) in patients with BRAF V600 wild-type tumors compared to 28% (95% CI, 26.4–30.36) in patients with BRAF V600 mutant tumors. The elevated LDH at the start of combined ICI was associated with a significantly reduced PFS compared to patients with normal LDH at the start (*p* < 0.001). The 2-year PFS was 34% (95% CI, 31.9–35.85) for patients with normal LDH and 8.5% (95% CI, 6.6–10.5) in the case of elevated LDH. PFS was significantly reduced when Protein S100 was elevated at the ICI start compared to those with normal S100 baseline (*p* = 0.047). The 2-year PFS was 26.5% (95% CI, 24.6–28.5) compared to 15% (95% CI, 13.9–16.91) in patients with increased S100 at the beginning of the combined ICI. Patients with AJCC stage M1c or M1d had a significantly reduced PFS compared to AJCC stage M0-M1b (*p* < 0.001). The 2-year PFS for patients with M1a/M1b was improved with 61.5% (95% CI, 59.7–63.6) compared to patients with M1c or M1d at ICI initiation. **Figure 2** displays the impact of patients’ characteristics on progression-free survival.

#### 3.1.4 Safety total cohort

In our study, 261 trAE had been reported in the patients’ files. In total, 33% of these were mild, 36% were moderate, 16% were grade 3, and 15% were grade 4 trAEs. The most common adverse event was fatigue, followed by colitis and pain. The most common grades 3 and 4 trAEs were colitis and hepatitis. There was one case of myocarditis (grade 4). In **Table 5**, the trAEs are reported.

**Table 5:** Treatment-related adverse effects total cohort.

	Mild (CTCAE 1)	Moderate (CTCAE 2)	Medically significant (CTCAE 3)	Life-threatening (CTCAE 4)	Death (CTCAE 5)	Total
Fatigue	42	4	1	0	0	47
Colitis	2	2	9	13	0	26
Pain	2	16	5	2	0	25
Eczema	5	11	2	2	0	20
Nausea	7	9	1	3	0	20
Pruritus	5	8	3	1	0	17
Vomiting	4	8	1	1	0	14
Infection/fever	1	10	1	1	0	13
Loss of appetite	8	2	1	0	0	11
Hepatitis	1	0	2	5	0	8
Pneumonitis	2	2	1	1	0	6
Arthralgia	0	5	1	0	0	6
Hypophysitis	0	1	3	2	0	6
Headache	1	4	0	1	0	6
Hypothyroidism	0	4	1	0	0	5
Pancreatitis	0	1	2	2	0	5
Obstipation	2	1	2	0	0	5
Hyperthyroidism	0	3	1	0	0	4
Neurologic disorders	1	1	1	1	0	4
Nephritis	0	0	2	2	0	4
Insomnia	2	0	0	0	0	2
Dizziness	1	1	0	0	0	2
Myositis	1	0	1	0	0	2
Dry mouth	1	0	1	0	0	2
Myocarditis	0	0	0	1	0	1
Total	88	93	42	38	0	261
%	33.22	35.63	16.09	14.56	0.00	

### 3.2 First-line cohort

A total of 40 patients started ICI in the first-line setting. The baseline patients’ characteristics of the first-line cohort are summarized in **Table 1** (right side).

#### 3.2.1 Efficacy first-line cohort

In **Table 2** (right side), the response rate to combined ICI with ipilimumab 1 mg/kg bw and nivolumab 3 mg/kg bw for the first-line cohort is reported in detail. The overall response rate was 32.5%, and the disease control rate was 50%.

#### 3.2.2 Overall survival first-line cohort

In the cohort of first-line patients, the median OS was not reached. The 2-year overall survival was 67% (95% CI, 51.3–82.3). Regarding the BRAF V600 mutation status, patients with BRAF V600 mutant tumors had a significantly better OS than patients with BRAF V600 wild-type tumors (*p* = 0.038). The median OS in BRAF V600 wild-type patients was 12 months (95% CI, 0–30.6 months). The 2-year OS was 58% (95% CI, 40.1–76.5) in BRAF V600 wild-type tumors. In contrast, all of the patients with BRAF V600 mutant tumors were still alive.

**Figure 3:**
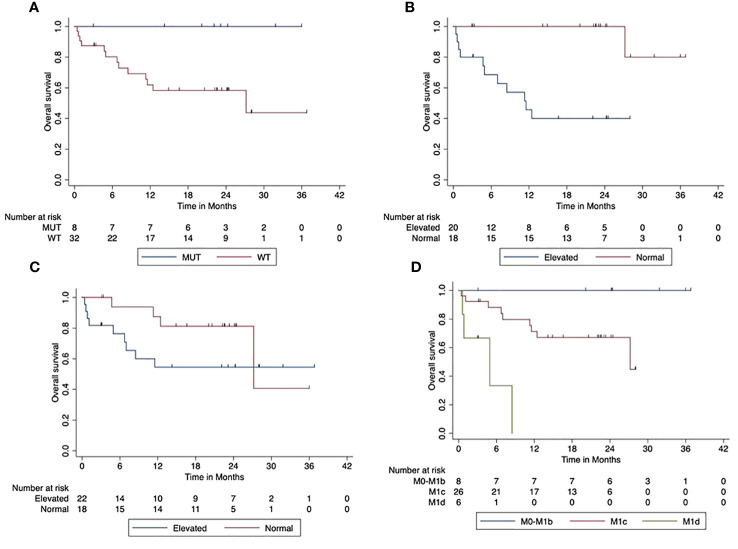
Impact of patient characteristics (first-line cohort) on overall survival. **(A)** BRAF mutant vs. wild-type tumors (*p* = 0.038). **(B)** LDH at start normal vs. elevated (*p* < 0.001). **(C)** Protein S100 at start normal vs. elevated (*p* = 0.154). **(D)** M-stage at start M0-M1b vs. M1c vs. M1d (*p* < 0.001).

Furthermore, OS was significantly (*p* < 0.001) lower in patients with elevated LDH at the beginning of the combined ICI. The median OS was 12 months (95% CI, 6.2–16.86). The 2-year OS was 100% in patients with normal LDH at the start of ICI compared to 40% (95% CI, 17.3–62.7) in patients with elevated LDH. There was a trend towards reduced OS in patients with elevated Protein S100 at the start of ICI compared to those with normal S100 (*p* = 0.154). The 2-year OS was 81% (95% CI, 62.1–100) in patients with normal S100 and 54.5% (95% CI, 32.2–76.8) in patients with elevated S100. Higher AJCC stages had a significantly negative impact on OS (*p* < 0.001). **Figure 3** shows the impact of patients’ characteristics of the first-line cohort on overall survival.

#### 3.2.3 Progression-free survival first-line cohort

**Figure 4:**
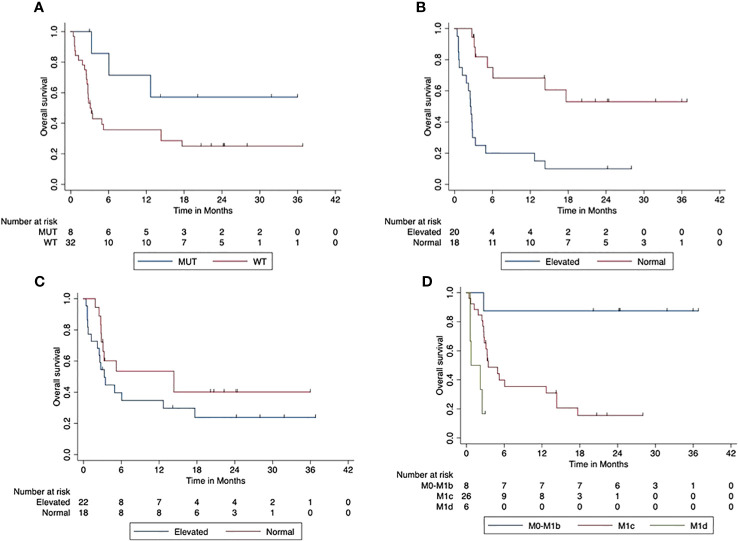
Impact of patient characteristics (first-line cohort) on progression-free survival. **(A)** BRAF mutant vs. wild-type tumors (*p* = 0.069). **(B)** LDH at start normal vs. elevated (*p* < 0.001). **(C)** Protein S100 at start normal vs. elevated (*p* = 0.165). **(D)** M-stage at start M0-M1b vs. M1c vs. M1d (*p* < 0.001).

The median PFS was 5 months (95% CI, 1.8–8.07). The 2-year PFS rate was 31% (95 CI%, 15.5–46.1). BRAF V600 mutated tumors were associated with a trend towards improved PFS compared to BRAF wild-type tumors (*p* = 0.069). The 2-year PFS was 57% (95% CI, 20.4–93.8) in patients with BRAF V600 mutant tumors and 25% (95% CI, 9.3–40.7) in patients with BRAF V600 wild-type tumors. The elevated LDH at the start of combined ICI was associated with a significantly reduced PFS compared to patients with normal LDH at the start (*p* < 0.001). The 2-year PFS was 53% (95% CI, 27.2–79.0) for patients with normal LDH and 10% (95% CI, 0–23.1) in the case of an elevated LDH. There was a trend likewise towards reduced PFS in patients with elevated Protein S100 at the start of ICI compared to those with normal S100 at the start (*p* = 0.165). The 2-year PFS rate was 40% (95% CI, 16.0–64.2) compared to 24% (95% CI, 5.0–42.6) in patients with increased S100 at the beginning of the combined ICI. Patients with higher AJCC stages (M1c or M1d) at the beginning of the combined ICI had a significantly reduced OS compared to patients with M0-M1b (*p* < 0.001). The 2-year PFS for patients with M1a/M1b was 87.5 (95% CI, 64.6–100), whereas that for patients starting with M1c was 15.5 (95% CI, 0–31.0) and that with M1d was 17% (95% CI, 0–46.5). **Figure 4** displays the impact of patients’ characteristics of the first-line cohort on progression-free survival.

#### 3.2.4 Safety first-line cohort

In **Table 6**, the trAE of the first-line cohort is described. In total, 154 trAEs were reported. The most common treatment-related adverse event was fatigue, which occurred in more than 60% of the patients. The second most frequent trAE was diarrhea in about a third of the patients. Pain, nausea, and eczema occurred in approximately 25% of the patients. More than 10% of the patients had hepatitis or hypophysitis, while for trAEs like dry mouth or myositis only very few patients suffered. There was no case of myocarditis. In our study, 33% of the patients suffered grade 3 or 4 trAE. There was no grade 5 trAE, thus no death from trAE. The most common grade 3 and grade 4 adverse event was diarrhea, followed by hepatitis and hypophysitis.

**Table 6:** Treatment-related adverse effects first-line cohort.

	Mild (CTCAE 1)	Moderate (CTCAE 2)	Medically significant (CTCAE 3)	Life-threatening (CTCAE 4)	Death (CTCAE 5)	Total
Fatigue	25	3	0	0	0	28
Colitis	1	1	7	7	0	16
Pain	1	8	2	1	0	12
Nausea	5	4	2	0	0	11
Eczema	3	6	1	1	0	11
Pruritus	2	4	2	1	0	9
Decreased appetite	7	1	0	0	0	8
Fever/infection	0	5	1	1	0	7
Vomiting	2	3	0	1	0	6
Hepatitis	1	0	1	4	0	6
Hypophysitis	0	1	2	2	0	5
Hypothyroidism	0	4	1	0	0	5
Obstipation	1	1	0	2	0	4
Hyperthyroidism	0	3	1	0	0	4
Nephritis	0	0	2	2	0	4
Pancreatitis	0	1	0	2	0	3
Headache	1	2	0	0	0	3
Arthralgia	0	2	1	0	0	3
Pneumonitis	1	0	1	0	0	2
Neurologic disorders	1	0	1	0	0	2
Dizziness	1	1	0	0	0	2
Insomnia	1	0	0	0	0	1
Dry mouth	0	0	1	0	0	1
Myositis	0	0	1	0	0	1
Myocarditis	0	0	0	0	0	0
Total	53	50	27	24	0	154
%	34.42	32.47	17.53	15.58	0	

## 4 Discussion

This study reports on advanced melanoma patients with flipped dosing of combined ipilimumab and nivolumab therapy in a real-world cohort with a large proportion of advanced M1c/d stages, elevated LDH levels, or challenging tumors such as mucosal or ocular melanoma. First of all, our results need to be compared to the CheckMate 511 trial. About 50% of our patients were older than 65 years, whereas in the CheckMate 511 study 64% of the patients were younger than 65 years, and approximately half of the patients in this trial were classified as metastatic stage M0/1a/1b at the beginning of the systemic treatment. In our cohort, only 20% of the patients were at one of these lower M stages; 80% had M1c/d. Concerning the LDH levels at the beginning of the combined ICI, in the CheckMate 511 cohort, 57% of the patients had normal LDH levels compared to 45% in our study population.

In addition, only 2% of the patients in the CheckMate 511 study had a history of brain metastasis compared to 15% patients with active brain metastasis in our population. Most of the patients in our cohort (67%) had BRAF V600 wild-type tumors, whereas in CheckMate 511 the BRAF V600 Status was nearly equal in both arms (48% vs. 43%). Only 13% of the patients in CheckMate 511 had had another systemic therapy before, which is markedly lower than in our population with approximately half of the patients having received another treatment before (10). In the CheckMate 511 study, the PD-L1 status was also raised. This was not the case in our study. PD-L1 is not determined as standard at the Tübingen Dermatology Clinic and could not be collected retrospectively, so no comparison can be made with the CheckMate 511 study (10). Concerning the results, the overall response rate in our cohort was 33%, and the disease control rate was 50%. Compared to the CheckMate 511 study, these rates are lower. In the CheckMate 511 study, the response rate was 46%, but in this study a much higher proportion of first-line patients had been included. Another reason could be the high proportion of BRAF V600 wild-type tumors in our cohort. These tumors are known to be not as responsive to ICI as BRAF V600 mutated tumors (11, 12). Another explanation could be the fact that we included also patients with mucosal or uveal melanoma and patients with active brain metastases, where the ICI response rates, in general, are lower (13–15). Furthermore, normal LDH and Protein S100 at the therapy start are known to be associated with an improved outcome (16).

When we look at the first-line patients of our cohort, the results of our real-world cohort correspond very well to the CheckMate 511 cohort—for example, the 2-year survival rate was approximately 66% in our cohort, and the median OS was not reached and is therefore very similar to that of the CheckMate 511. In the CheckMate 511 study, the 2-year survival rate was 65%, and the median OS was not reached as well (10). Particularly noteworthy is the result of the side effects observed. The most common side effects in our study were diarrhea, fatigue, and pruritus, which are very similar to other studies (17–19).

Several studies report that ICI are commonly associated with gastrointestinal, endocrinological, and dermatologic adverse events, while the cardiotoxicity is very raw (3, 5, 10, 20). This distribution of side effects corresponds to what we found in our cohort. In our study, 82.5% of the patients suffered with any treatment-related side effect. In the CheckMate 511 study, it was approximately 86%. The grade 1 and grade 2 trAEs are not further broken down in the CheckMate 511 study.

However, the grades 3 and 4 adverse events in our cohort occurred less frequently compared to the combined ipilimumab and nivolumab standard dose of the CheckMate 067 study. The frequency of grades 3 and 4 adverse events with 33% in our cohort corresponds well to the reported 34% in patients of the CheckMate 511 study. In that trial, the liver and colon were the most frequently involved organs with hepatitis and colitis (21). Therefore, our results fit well not only in terms of the absolute percentages but also in terms of involved organs with those of Lebbé et al. Although the rate of trAE is much lower with the flipped dosing of ipilimumab and nivolumab, patients still need to be closely monitored (22).

Nevertheless, this retrospective study has a few limitations. Adverse events were collected in the patients’ files retrospectively as documented by the treating physicians. This documentation is almost certainly complete if the trAE resulted in specific procedures such as discontinuation of ICI or treatment initiation with immunosuppressive drugs. If the treatment regime was continued unchanged either because the AE was so mild or because it was still uncertain whether it was an AE at all, it may have happened that the documentation was not as thorough. The results of our study should be confirmed in other cohorts with more real-world melanoma patients. On the other side, we have the strength of a large cohort treated by one single center with the same staging and laboratory surveillance procedures for all patients—for example, all patients were discussed by the same tumor boards and the same radiologists. This ensures a high-quality standard despite a real-world setting.

## 5 Conclusion

In this study, we found that combined immune checkpoint inhibition with ipilimumab and nivolumab in flipped dosing is an effective and safe treatment schedule in advanced melanoma in daily clinical use. The percentage of grade 3 or 4 side effects with the flipped dosing corresponds well to that of the CheckMate 511 study population that had a significantly lower rate compared to the standard dosing. In addition, the overall survival and progression-free survival for the first-line patients were equal in our study cohort compared to the cohort of CheckMate 511. For this reason, we consider the flipped dosing to be a regimen that can be used in everyday clinical practice, especially in patients who have the greatest concerns about potential side effects with the standard dose. Nevertheless, it has to be considered that flipped dosing has not been approved for melanoma and should be reserved for selected cases.

## Data availability statement

The raw data supporting the conclusions of this article will be made available by the authors, without undue reservation.

## Ethics statement

The study was conducted in accordance with the Declaration of Helsinki and approved by the Institutional Review Board, project number 899/2021BO2, by the Ethics Committee of Eberhard-Karls-University of Tübingen from 14.12.2021.

## Author contributions

CN: Conceptualization, Methodology, Resources, Writing – original draft, Writing – review & editing, Data curation, Formal Analysis, Investigation, Visualization. TA: Writing – review & editing. UL: Writing – review & editing. LF: Writing – review & editing. AF: Writing – review & editing, Conceptualization, Methodology, Project administration, Resources, Supervision, Writing – original draft.

## References

[1] Wolchok JD Chiarion-Sileni V Gonzalez R Rutkowski P Grob JJ Cowey CL. Overall survival with combined nivolumab and ipilimumab in advanced melanoma. New Engl J Med (2017) 377(14):1345–56. doi: 10.1056/NEJMoa1709684 PMC5706778 28889792

[2] Hodi FS Chiarion-Sileni V Gonzalez R Grob JJ Rutkowski P Cowey CL. Nivolumab plus ipilimumab or nivolumab alone versus ipilimumab alone in advanced melanoma (CheckMate 067): 4-year outcomes of a multicentre, randomised, phase 3 trial. Lancet Oncol (2018) 19(11):1480–92. doi: 10.1016/S1470-2045(18)30700-9 30361170

[3] Wolchok JD Chiarion-Sileni V Gonzalez R Grob JJ Rutkowski P Lao CD. Long-term outcomes with nivolumab plus ipilimumab or nivolumab alone versus ipilimumab in patients with advanced melanoma. J Clin Oncol (2022) 40(2):127–37. doi: 10.1200/JCO.21.02229 PMC8718224 34818112

[4] Schadendorf D Wolchok JD Hodi FS Chiarion-Sileni Gonzalez R Rutkowski P. Efficacy and safety outcomes in patients with advanced melanoma who discontinued treatment with nivolumab and ipilimumab because of adverse events: A pooled analysis of randomized phase II and III trials. J Clin Oncol (2017) 35(34):3807–14. doi: 10.1200/JCO.2017.73.2289 PMC5791828 28841387

[5] Choi J Lee SY. Clinical characteristics and treatment of immune-related adverse events of immune checkpoint inhibitors. Immune Netw (2020) 20(1):e9. doi: 10.4110/in.2020.20.e9 32158597 PMC7049586

[6] Weber J Mandala M Del Vecchio M Gogas HJ Arance AM Cowey CL. Adjuvant nivolumab versus ipilimumab in resected stage III or IV melanoma. New Engl J Med (2017) 377(19):1824–35. doi: 10.1056/NEJMoa1709030 28891423

[7] Hogg D Chapman PB Sznol M Lao CD Gonzalez R Daniels GA. Overall survival (OS) analysis from an expanded access program (EAP) of nivolumab (NIVO) in combination with ipilimumab (IPI) in patients with advanced melanoma (MEL). J Clin Oncol (2017) 35(Suppl 15):9522–2. doi: 10.1200/JCO.2017.35.15_suppl.9522

[8] Hofmann L Forschner A Loquai C Goldinger SM Zimmer L Ugurel S. Cutaneous, gastrointestinal, hepatic, endocrine, and renal side-effects of anti-PD-1 therapy. Eur J Cancer (2016) 60:190–209. doi: 10.1016/j.ejca.2016.02.025 27085692

[9] Hodi FS Chesney J Pavlick A Robert C Grossmann KF McDermott DF. Combined nivolumab and ipilimumab versus ipilimumab alone in patients with advanced melanoma: 2-year overall survival outcomes in a multicentre, randomised, controlled, phase 2 trial. Lancet Oncol (2016) 17(11):1558–68. doi: 10.1016/S1470-2045(16)30366-7 PMC5630525 27622997

[10] Lebbé C Meyer N Mortier L Marquez-Rodas I Robert C Rutkowski P. Evaluation of two dosing regimens for nivolumab in combination with ipilimumab in patients with advanced melanoma: results from the phase IIIb/IV checkMate 511 trial. J Clin Oncol (2019) 37(11):867–75. doi: 10.1200/JCO.18.01998 PMC6455714 30811280

[11] Moser JC Chen D Hu-Lieskovan S Grossmann KF Patel S Colonna SV. Real-world survival of patients with advanced BRAF V600 mutated melanoma treated with front-line BRAF/MEK inhibitors, anti-PD-1 antibodies, or nivolumab/ipilimumab. Cancer Med (2019) 8(18):7637–43. doi: 10.1002/cam4.2625 PMC6912019 31677253

[12] van Not OJ Blokx WAM van den Eertwegh AJM de Meza MM Haanen JB Blank CU. BRAF and NRAS mutation status and response to checkpoint inhibition in advanced melanoma. JCO Precis Oncol (2022) 6:e2200018. doi: 10.1200/po.22.00018 36130145

[13] Yde SS Sjoegren P Heje M Stolle LB. Mucosal melanoma: a literature review. Curr Oncol Rep (2018) 20(3):28. doi: 10.1007/s11912-018-0675-0 29569184

[14] Wessely A Steeb T Erdmann M Heinzerling L Vera J Schlaak M. The role of immune checkpoint blockade in Uveal melanoma. Int J Mol Sci (2020) 21(3):879. doi: 10.3390/ijms21030879 32013269 PMC7037664

[15] Henon C Remon J Hendriks LE. Combination treatments with immunotherapy in brain metastases patients. Future Oncol (2020) 16(23):1691–705. doi: 10.2217/fon-2020-0156 32412817

[16] Weide B Elsässer M Büttner P Pflugfelder A Leiter U Eigentler TK. Serum markers lactate dehydrogenase and S100B predict independently disease outcome in melanoma patients with distant metastasis. Br J Cancer (2012) 107(3):422–8. doi: 10.1038/bjc.2012.306 PMC3405231 22782342

[17] Darnell EP Mooradian MJ Baruch EN Yilmaz M Reynolds KL. Immune-related adverse events (irAEs): diagnosis, management, and clinical pearls. Curr Oncol Rep (2020) 22(4):39. doi: 10.1007/s11912-020-0897-9 32200442

[18] Kähler KC Hassel JC Heinzerling L Loquai C Mössner R Ugurel S. Management of side effects of immune checkpoint blockade by anti-CTLA-4 and anti-PD-1 antibodies in metastatic melanoma. J Dtsch Dermatol Ges (2016) 14(7):662–81. doi: 10.1111/ddg.13047 27373241

[19] Brunot A Grob JJ Jeudy G Grange F Guillot B Kramkimel N. Association of anti-programmed cell death 1 antibody treatment with risk of recurrence of toxic effects after immune-related adverse events of ipilimumab in patients with metastatic melanoma. JAMA Dermatol (2020) 156(9):982–6. doi: 10.1001/jamadermatol.2020.2149 PMC7364335 32667663

[20] Choi JS Chandra S. Targeted therapy for melanomas without BRAF V600 mutation. Curr Oncol Rep (2022) 24(12):1873–81. doi: 10.1007/s11912-022-01306-z 36435868

[21] Reddy HG Schneider BJ Tai AW. Immune checkpoint inhibitor-associated colitis and hepatitis. Clin Transl Gastroenterol (2018) 9(9):180. doi: 10.1038/s41424-018-0049-9 30228268 PMC6143593

[22] Weinstein A Gordon RA Kasler MK Burke M Ranjan S Hodgetts J. Understanding and managing immune-related adverse events associated with immune checkpoint inhibitors in patients with advanced melanoma. J Adv Pract Oncol (2017) 8(1):58–72. doi: 10.6004/jadpro.2017.8.1.5 29900017 PMC5995532

